# Prognostic value of programmed cell death ligand-1 expression in breast cancer: A meta-analysis

**DOI:** 10.1097/MD.0000000000023359

**Published:** 2020-12-04

**Authors:** Yingzi Zhang, Jiao Tian, Chi Qu, Zhenrong Tang, Yu Wang, Kang Li, Yuan Yang, Shengchun Liu

**Affiliations:** a Department of Endocrine Breast Surgery; b Department of Cardiovascular Medicine, the First Affiliated Hospital of Chongqing Medical University, Chongqing, China.

**Keywords:** breast cancer, disease free survival, meta-analysis, overall survival, programmed cell death ligand-1

## Abstract

**Background:**

The correlation between programmed cell death-ligand 1 (PD-L1) which may affect T cell to form the immune tolerance and breast cancer (BC) still maintains to be uncovered. This meta-analysis was about to explore PD-L1 expression as well as its prognostic role in BC.

**Methods:**

First of all, we performed 3 databases: PubMed, Embase, and Web of Science to explore publications between January of 2015 and January of 2020. Strict inclusion and exclusion criteria were conducted: immunohistochemistry shall be used to detect target molecule expression and at least 1 survival indicator and related data we need should be included. The hazard ratio and 95% confidence interval were pooled related with survival as well as clinicopathological parameters. The effects of PD-L1 in differed aspects like sample size and age of each cohort were demonstrated by subgroup analyses as well as sensitivity analyses which may complain the potential source of heterogeneity. *P* < .05 indicates factors were charge of the heterogeneity of prognosis. Begg and Egger tests were used to identify publication bias.

**Results:**

We identified 12 studies containing a blanket of 4336 patients with BC for whom PD-L1 positive tumor cells were related with higher tumor stage, lymph node metastasis, estrogen receptor negativity, human epidermal growth factor receptor 2 positivity, luminal B and triple negative BC molecular subtype and high nuclear-associated antigen Ki- 67 expression. Meanwhile, compared to patients with PD-L1 negative expression, PD-L1 positivity associated with worse overall survival (Hazard ratio [HR]:1.43; 95% CI:0.98–2.10; *P* < .001) and might have no obvious tight connection with disease free survival (HR:1.40; 95% CI:1.11–1.78; *P* = .101) and recurrence free survival (HR:2.36; 95% CI:1.04–5.34; *P* = .145). The outcome of the meta-analysis was confirmed to be credible by sensitivity analysis. Publication bias was not existed indicated (*P* = .640).

**Conclusion:**

Positive PD-L1 expression has a worse clinical outcome in patients with BC demonstrated by our meta-analysis. Being urgent to catch attention to the role of PD-L1 in BC, it may be considered as prognostic marker of immune microenvironment for improving therapy efficacy.

## 1 Introduction

Breast cancer (BC) is still the leading type of malignant tumors that affects the prognosis of women with high mortality rate so far. Treatment guidelines have been followed up and improved by various researchers, especially in terms of aspect like tumor microenvironment. Recently, survival benefits have been suggested to relate with the performance of chemotherapy, adjuvant therapies, and receptor target therapies among various clinical trials, in which researchers still acclaimed that there maintain metastasis and recurrence resulting in death.^[1]^ More effective treatment is about to be conducted, while tumor progression and consequently death have been reported to keep increasing among BC patients so far.^[2]^ Reports show us that hormone receptor positive BC carried out the increasing tendency of that, while the incidence rate of hormone receptor negative BC goes reverse in whole text.^[3]^ The prognostic elements in BC have been investigated for decades; however, prognostic factors other than stage and performance status are still controversial. Previous studies have inferred clinicopathological features such as larger tumor size, hormone receptor status, the presence of lymph node involvement, staining extent of nuclear-associated antigen Ki- 67 (Ki-67), as adverse distinguished prognostic traits in BC.

ln theory, heterogeneity always exists when individuals in specific ethic are identified as patients who have received similar treatment. Meanwhile, the presented anatomic staging system is not comprehensive enough which also can carry out the differed clinical outcome of those selected patients.

Except for TNM stage, these factors only can be evaluated after surgical operation. Therefore, there is significant attraction in investigating noninvasive and readily accessible pretreatment variables to evaluate survival outcome in BC. Inflammatory treatment might play an attention-catching role in tumor progression demonstrated by latest reports.

Many complicated mechanisms of immune microenvironment and tumor could be responsible by the immune tolerance causing by programmed cell death ligand-1 (PD-L1) and its related B7 family. Also, benefit has been approached for patients with BC with PD-L1 immune blockades, leaving a message that PD-L1 shall be indicative of prognostic molecule.^[4,5]^

In the only reported phase 3 trial, the combination of atezolizumab and nabpaclitaxel conferred a nonstatistically significant overall survival (OS) benefit compared to nab-paclitaxel alone in unselected triple negative breast cancer (TNBC) patients. Intriguingly, the diversity in OS was significant in the PD-L1+ subgroup of patients, suggesting that the potential clinical utility of PD-L1 expression.^[6,7]^ In BC, quite a few studies have currently suggested that positive PD-L1 BC was related with poorer OS,^[8–10]^ but other studies could not verify this finding.^[11,12]^ However, according to provided results, this eye on the value of PD-L1 in BC still did not reach a consensus that further validation is urgently awaited.

As the spread knowing, to overcome the limitation of various variates causing the heterogeneity from cohorts, meta-analysis is a powerful statistic tool, which also may generate the comprehensive and convincible data to explain the ultimate clinical relationship.

## 2 Materials and methods

### 2.1 Ethics statement

The present study was conducted guiding by Preferred Reporting Items for Systematic Reviews and Meta-Analyses (PRISMA). Due to based literature, ethical clearance is not required.^[13]^

### 2.2 Search strategy

On February 16 of 2020, a literature selection of studies was performed within recent 5 years limitation through well-known databases: PubMed, Embase, Web of science. All terms used were as follows: “breast neoplasms or BC or breast carcinoma or Breast Tumor or Mammary Cancer or Malignant Neoplasm of Breast or Human Mammary Neoplasm or Cancer of Breast,” and “Programmed Cell Death 1 Ligand 1 or B7-H1 Immune Costimulatory Protein or B7 H1 Immune Costimulatory Protein or PD-L1 Costimulatory Protein or Costimulatory Protein, PD-L1 or PD L1 Costimulatory Protein or CD274 Antigen.” Two independent authors reviewed the search results, respectively.

### 2.3 Inclusion and exclusion criteria

Inclusion criteria followed by:

(1) selected patients with BC were confirmed by pathological standard method;
(2) studies in which anti-PD-L1 antibody for the immunohistochemistry was collected;
(3) studies in which one or more clinical survival outcomes were reported;
(4) core needle biopsy method was used or specimens directly were resected after surgery.

Exclusion criteria followed by:

(1) comments, reviews, abstracts or non-BC cohorts;
(2) non-English articles;
(3) incomplete data for survival with hazard ratio and 95% confidence interval;
(4) duplicate studies;
(5) patients who had received neoadjuvant or adjuvant treatment or enrolled PD-L1 inhibitor clinical trial.

Criteria were strictly carried out. EndNote software (version X9) and manual screening method were used to achieve the most suitable and complete studies for analysis. To achieve the consensus, final case was triggered by a third author in terms of disagreement.

### 2.4 Data extraction and quality assessment

Our research included author of cohort, year, nation, sample size, patient age, PD-L1 antibody information, PD-L1 positivity, detection standard, follow-up, Hazard ratio (HR) and 95% CI and clinical endpoints. Positive or high expression is defined as PD-L1+, left was classified as PD-L1-. All eligible studies were retrospective. Newcastle-Ottawa Scale (NOS) system consists of three parts: selection (0–4 points), comparability (0–2 points), and outcome assessment (0–3 points). The Newcastle-Ottawa Scale (NOS) score of 6 were assigned as high-quality studies.

### 2.5 Statistical analysis

OS, disease free survival (DFS) and recurrence free survival (RFS) were defined as endpoints of survival outcomes in this study. The time from the first diagnosis to death for any reason is defined for OS. DFS is defined as the interval from the date of surgery to the first observation of recurrence. RFS, the time from treatment initiation to recurrence at any site. Mantel–Haenszel method was performed to analyze pooled HR with 95% CI to all extent. The pooled results of those clinical prognostic outcomes were indicated by forest plots. Post this, to address heterogeneity, tests were conducted by Cochran *Q* and Higgins I-squared method. A fixed-effect model might be chosen to obtain precise results with insignificant heterogeneities, a random-effect model was utilized otherwise. *I*^2^ < 25%, *I*^2^ = 25% to 50%, and *I*^2^ > 50% denote no heterogeneity, low heterogeneity, and extreme heterogeneity separately. *P* < .1 or I-squared >50% was indicative of remarkable heterogeneity^[14]^; Sensitivity analysis is a tool that analyzes whether the combined effect amount after the exclusion of the included studies varied from the previous total effect amount. If the 2 HRs are very different, it means that the excluded study may explain the resource of heterogeneous.^[15]^ Subgroup analyses were applied for the same purpose.^[14]^ Publication bias was assessed by visual inspection of Begg plot and the possibility of publication bias was conducted by Egger test,^[16,17]^ which should be taken into considered when *P* < .05. Review Manager 5.3, STATA version 15.0, and IBM Statistics 23 were used for analyses. McNemar and Pearson Chi-square tests were adopted to identify association between clinical parameter and expression of PD-L1. A 2-sides *P* < .05 was considered statistically significant.

## 3 Results

### 3.1 Study characteristics

**Figure 1:**
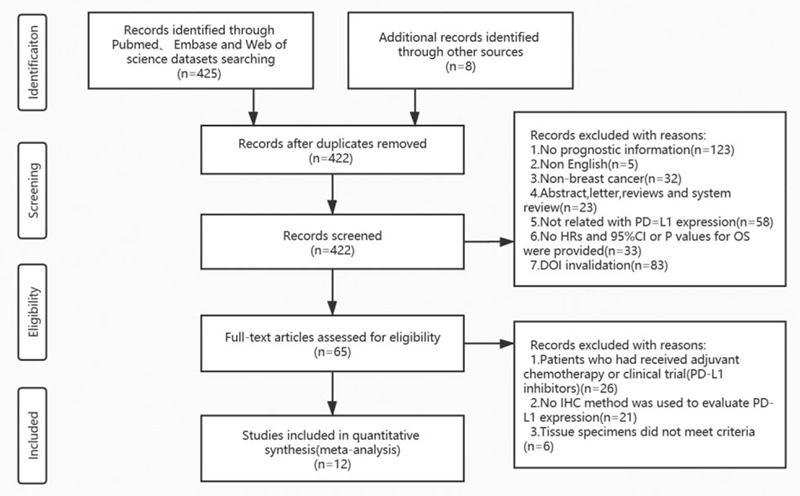
Flow chart of the included studies.

Four hundred thirty-three literature were researched in total and the selection process was summarized in Figure 1. After manual screening by 2 authors, 368 articles were ruled out because they were letters, duplicates, reviews, abstracts, studies on other tumors, or no data related HRs and 95%CIs. After screening 65 complete records, we also eliminated 53 articles because patients were not eligible for inclusion in those studies who had already received neoadjuvant or adjuvant chemotherapy or enrolled any clinical trial before surgery, or not using IHC method to evaluate PD-L1 expression. At last, 12 articles meeting criteria were included for this meta-analysis.^[9,10,12,18–26]^Table 1 shows the basic characteristics like year of cohort, sample size, follow-up, NOS score et al. Table 2 demonstrated the correlation of PD-L1 expression among various clinicopathological features, like hormone status and tumor-related classification and so on. Including reports were published between 2014 and 2018. In this study 4336 patients with BC included, we identified 1279 cases as luminal A BC, 1044 patients were classified as luminal B, 360 individuals suffered HER-2 rich, 1236 women belonged to TNBC type. Multivariate analyses were performed in these included studies to address prognostic outcomes including DFS and OS in most of cohorts. And immunohistochemistry method was conducted shown in Table 1. In total, 12 studies had estimated in tumor cells expression of PD-L1^[9,10,12,18–26]^ and 5 studies evaluated expression of PD-L1 in both tumor and immune cells.^[19,22–24,26]^ Also, different study owns different criteria for PD-L1 cut-off values which were summarized in Table 1.

**Table 1:** Characteristics of the studies included in the meta-analysis.

Study cohort	Year	Country	N	Age (yrs; median and range)	Type of study	PD-L1 Antibody used	PD-L1+(%)	Detection standard	Study end-points	HR	Follow-up, median in months	NOS score
António Polónia	2017	Spain	440	60.0(28–92)	Retrospective	clone SP142(R)	28 (6.4%)	membranous/cytopl-asmic staining≥1%	OS	R (M)	120 (1–120)	6
Xiaoxian Li	2016	USA	136	NA	Retrospective	clone NAT105 (M)	14 (10.3%)	H-score≥5	OS/DFS	R (M)	36-144	6
Julia Y. S. Tsang	2017	China	1091	54.5 ± 12.7 (22–94)	Retrospective	NA	295 (27.0%)	Mean immunoscore (staining intensity)	OS/DFS	R (M)	63 (1–210)	7
Jing He	2018	USA	68	48.0 (23–75)	Retrospective	clone 28–8 (R)	25 (36.8%)	Mean immunoscore (staining intensity)	OS/DFS	R (M/U)	48 (23–75)	8
Ming Li	2018	China	101	51 (27–74)	Retrospective	CST, 13,684 (R)	39 (38.61%)	H-score≥5	DFS	R (M/U)	49.03 (10.97–94.27)	7
Hitomi Mori	2017	Japan	284	59.6	Retrospective	E1L3N (R)	103 (41.5%)	PD-L1 expression≥50%	OS/RFS	R (M/U)	68 (2–150)	6
Quirine F. Manson	2018	Netherlands	106	53	Retrospective	clone sp263 (R)	14 (13.2%)	H-scores>0	OS	R (M/U)	61.2 (15.6–310.8)	6
Sang Byung Bae	2016	Korea	465	52.3 (24–81)	Retrospective	E1L3N (R)	63 (13.5%)	H-score ≥100	OS/DFS	R (M)	41 (1–158)	8
In Hae Park	2015	Korea	333	47 (28–78)	Retrospective	Abcam (R)	163 (48.9%)	H-score≥2+-3+	OS/DFS	R (U)	117.6 (4.8–153.6)	8
Rhiannon K Beckers	2015	Australia	161	57 (28–89)	Retrospective	E1L3N (R)	123 (76.4)	H-score≥100	OS/CSS	R (U)	55 (0–213)	7
S. Muenst	2014	Switzerland	650	64 (27–101)	Retrospective	Abcam (R)	152 (23.4%)	H-score ≥100	OS	R (M/U)	65 (1–174)	6
Zhenhua Li	2016	China	501	53 (29–83)	Retrospective	ab58810 (R)	231 (46.1%)	H-score ≥100	OS/RFS	R (M/U)	64 (1–80)	8

USA = United States of America, N = number of patients, NA = not applicable, R/M = rabbit/mouse, OS = overall survival, DFS = disease free survival, RFS = recurrence free survival, TNBC = triple negative breast cancer, R(M) = the HR come from multivariate analysis, R(U) = the HR comes from univariate analysis, NOS score = The Newcastle-Ottawa Scale (NOS) score.

**Table 2:** Correlation between PD-L1 expression and clinicopathological parameters.

	PD-L1(-)%	PD-L1(+)%	*P* value
Age (yrs)			0.051
≤50	333 (57.3)	248 (42.7)	
>50	433 (62.4)	260 (37.6)	
tumor size (cm)			0.466
≤2	246 (50.8)	238 (49.2)	
>2	322 (53.0)	285 (47)	
Histologic grade			0.779
I	424 (74.1)	148 (25.9)	
II	1110 (73.4)	402 (26.6)	
III	977 (72.6)	368 (27.4)	
Tumor stage			**0.015**
PT1	662 (78.3)	184 (21.7)	
PT2	875 (78.1)	246 (21.9)	
PT3	84 (71.8)	33 (28.2)	
PT4	70 (66.0)	36 (34.0)	
Lymph node metastasis			**0.007**
(–)	1007 (72.1)	390 (27.9)	
(+)	802 (67.2)	391 (32.8)	
ER status			**.000**
(–)	897 (73.2)	328 (26.8)	
(+)	1741 (80.2)	429 (19.8)	
PR status			.167
(–)	950 (75.5)	309 (24.5)	
(+)	972 (73.1)	358 (26.9)	
HER2 status			**0.030**
(–)	2122 (73.7)	758 (26.3)	
(+)	508 (69.7)	221 (30.3)	
Molecular subtype			**0.043**
Luminal A	762 (75.3)	250 (24.7)	
Luminal B	570 (70.3)	241 (29.7)	
Her2 rich	209 (77.4)	61 (22.6)	
TNBC	346 (73.2)	127 (26.8)	
Ki-67 expression			**0.000**
Low	1283 (78.5)	352 (21.5)	
High	951 (69.0)	428 (31.0)	

ER = estrogen receptor, PR = progesterone receptor, T = tumor, *P* < .05: statistically significant.

### 3.2 PD-L1 expression and clinicopathological features

Table 2 clearly summarized the correlation between PD-L1 positive in tumor cells and clinicopathological parameters. Ten (83.3%) studies^[9,10,12,18,20–25]^ had reported PD-L1 positivity in different ways. PD-L1 + associated with high tumor stage (stage 1 vs 2 vs 3 vs 4, 21.7% vs 21.9% vs 28.2% vs 34.0%, *P* = .015), lymph node metastasis (positive vs negative, 32.8% vs 27.9%, *P* = .007), estrogen receptor (ER) negativity (ER positive vs ER negative, 19.8% vs 26.8%, *P* = .000), human epidermal growth factor receptor 2 (HER2) positivity (HER2+ vs HER2, 30.3% vs 26.3%, *P* = .03), luminal B and TNBC molecular subtype (luminal A 24.7%, luminal B 29.7%, HER2-rich 22.6%, TNBC 26.8%, *P* = .043) and higher Ki-67 expression (low expression vs high expression, 21.5% vs 31.0%, *P* = .000). Meanwhile, we were not given the strong hint with the association between expression of PD-L1 and age (*P* = .051), tumor size (*P* = .466), histologic grade (*P* = .779) as well as progesterone receptor status (*P* = .167).

### 3.3 PD-L1 expression and patient survival

Related data in present study were carried out from the collected publications including figures and table numeric data to calculate total HRs and 95% CIs about PD-L1 expressed in tumor cells.^[27]^

**Figure 2:**
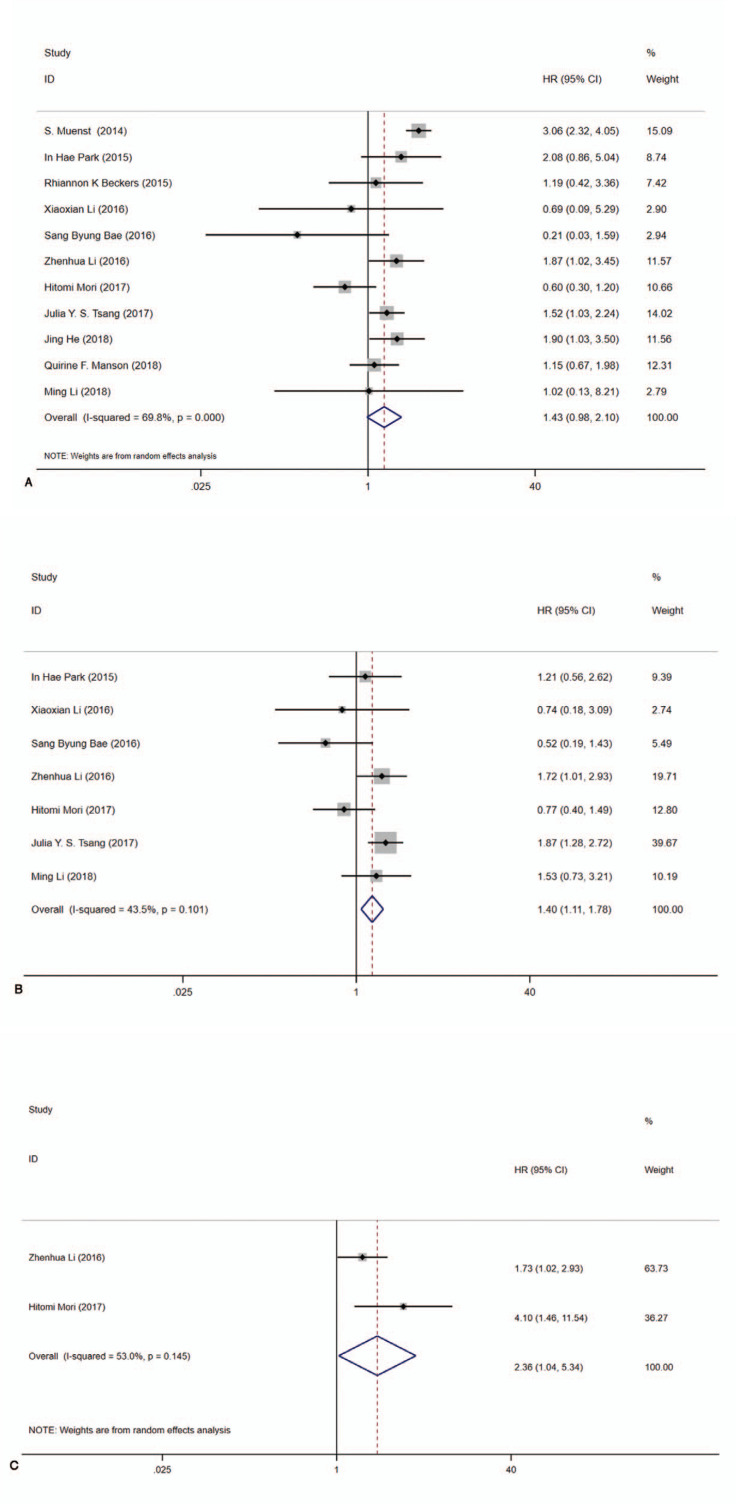
Forest plots of hazard ratios (HR) for survival based on PD-L1 expression. A, OS (pooled HR 1.43, 95%CI = 0.98–2.10, *I*^2^ = 69.8%, Cochrane Q, *P* < .000). B, DFS (pooled HR 1.40, 95% CI = 1.11–1.78, *I*^2^ = 43.5%, Cochrane Q, *P* = .101). C RFS (pooled HR 2.36, 95% CI = 1.04–5.34, *I*^2^ = 53.0%, Cochrane Q, *P* = .145). CI = confidence interval, OS = overall survival, DFS = disease-free survival, RFS = recurrence-free survival.

For OS, altogether 11 studies^[9,10,12,19–26]^ reported OS data with precise HR and 95%CIs. Significant heterogeneity existed (*I*^2^ = 69.8%, Cochrane Q) among extracted studies. As a result, pooled data suggested that PD-L1 expression was implicated in shorter OS with a random model (pooled HR:1.43, 95%CI:0.98–2.10, *P* < .001) (Fig. 2A). For DFS, there were seven studies in which data we need were displayed^[10,12,19,20,22,23,25]^ and low heterogeneity was existed (*I*^2^ = 43.5%, Cochrane Q). Pooled result by fixed model showed that PD-L1 expression had no profound impact on DFS in PD-L1 positive patients with BC (pooled HR:1.40, 95% CI:1.11–1.78, *P* = .101) (Fig. 2B). Only 2 studies^[10,23]^ provided RFS data and obviously significant heterogeneity existed (*I*^2^ = 53.0%, Cochrane Q). According to the given explicit data, statistical difference was not observed between PD-L1 expression and RFS in PD-L1 positive BC patients (pooled HR:2.36, 95% CI:1.04–5.34, *P* = .145) (Fig. 2C).

### 3.4 Sensitivity analysis

Sensitivity analysis was selected to determine whether omitting every study turning out a significant difference. Accordingly, pooled results were achieved after leaving out each study in turn, no specific study significantly changed the overall HRs, which means that the credible outcomes were indicated (Supplemental Digital Content, Fig. S1).

### 3.5 Subgroup analysis

**Figure 3:**
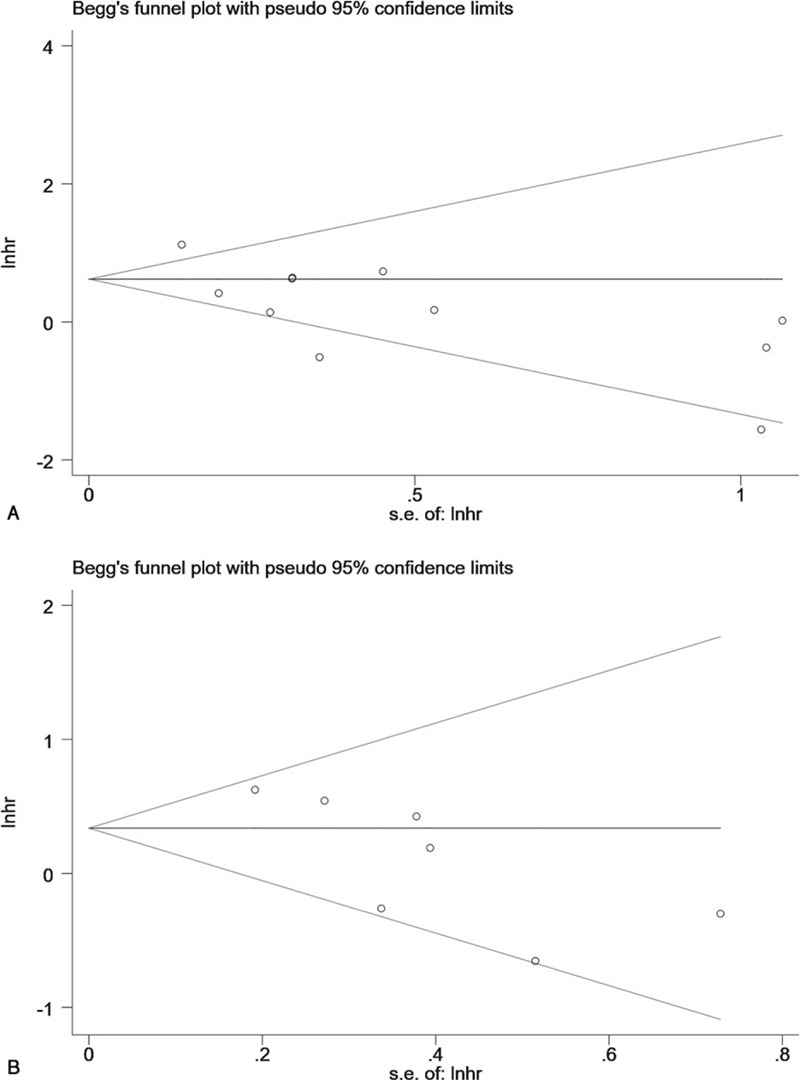
Begg test for all included studies. A, Overall survival (*P* = .640) B, Disease free survival (*P* = .072).

Subgroup analyses evaluated OS by a random-effects model and results were summarized in Table 3. Our results indicated the pooled HR was 1.13 (95%CI:0.75–1.71, *I*^2^ = 34.30%, *P* = .154) for sample size ≤500 and 2.11 (95%CI: 1.28–3.46, *I*^2^ = 77.40%, *P* = .012) for sample size >500. In addition, subgroup analysis was performed by age (≤50 and >50), Univariate analysis and multivariate analysis (U/M), NOS score (Fig. 3). Meantime, a fixed-effects model was adopted to estimate in terms of DFS. The pooled HR was 0.96 (95%CI: 0.66–1.39, *I*^2^ = 0.000%, *P* = .428) for sample size ≤500 and 1.82 (95%CI: 1.34–2.47, *I*^2^ = 0.000%, *P* = .806) for sample size >500. Also, subgroup analysis was performed by age (≤50 and >50), Univariate analysis and Multivariate analysis (U/M), NOS score (Supplemental Digital Content, Fig. S2). Besides, we used meta regression to identify triggers of heterogeneity that contributed to differences in clinical outcomes, but unfortunately, several factors we examined were not the main causes of the heterogeneity (Supplemental Digital Content, Fig. S3), in which *P* > .05.

**Table 3:** Summary of the meta-analysis results.

			Random-effects model		Fixed-effects model		Heterogeneity	
Analysis	N	References	HR (95%CI)	*P*	HR (95%CI)	*P*	*I*^2^	Ph
OS	11	9,10,12,19,20,21,22,23,24,25,26	1.43 (0.98–2.10)	.067	1.86 (1.56–2.21)	.000	69.80%	0.000
Subgroup1:sample size <500	8	12,19,21,22,23,24,25,26	1.13 (0.75–1.71)	.551	1.17 (0.87–1.58)	.301	34.30%	0.154
sample size ≥500	3	9,10,20	2.11 (1.28–3.46)	.003	2.34 (1.89–2.90)	.000	77.40%	0.012
Subgroup2: age <50	2	12,21	1.96 (1.18–3.24)	.009	1.96 (1.18–3.24)	.009	0.00%	0.869
Age ≥50	7	9,10,20,22,23,25,26	1.32 (0.81–2.14)	.269	1.86 (1.55–2.24)	.000	78.20%	0.000
Subgroup3: univariate analysis	3	12,23,24	1.08 (0.58–2.03)	.803	1.05 (0.72–1.55)	.795	59.20%	0.000
Multivariate analysis	8	9,10,19,20,21,22,25,26	1.70 (1.13–2.54)	.01	2.15 (1.77–2.61)	.000	60.30%	0.014
Subgroup4: NOS score =6	4	9,19,23,24	1.23 (0.50–3.05)	.655	2.10 (1.66–2.64)	.000	88.30%	0.000
NOS score= 7	3	20,22,26	1.46 (1.02–2.09)	.04	1.46 (1.02–2.09)	.040	69.80%	0.000
NOS score= 8	4	10,12,21,25	1.70 (1.03–2.81)	.037	1.78 (1.21–2.60)	.003	33.10%	0.214
DFS	7	10,12,19,20,22,23,25	1.26 (0.89–1.78)	.195	1.40 (1.11–1.78)	.005	43.50%	0.101
Subgroup1:sample size <500	5	12,19,22,23,25	0.96 (0.66–1.39)	.832	0.96 (0.66–1.39)	.832	0.00%	0.428
sample size ≥500	2	10,20	1.82 (1.34–2.47)	0	1.82 (1.34–2.47)	.000	0.00%	0.806
Subgroup2: age <50	1	12	1.21 (0.56–2.62)	.628	1.21 (0.56–2.62)	.628	NA	NA
Age ≥50	5	10,20,22,23,25	1.28 (0.83–1.96)	.259	1.45 (1.13–1.87)	.004	58.50%	0.047
Subgroup3: univariate analysis	2	12,19	1.08 (0.55–2.13)	.818	1.08 (0.55–2.13)	.818	0.00%	0.553
Multivariate analysis	5	10,20,22,23,25	1.28 (0.83–1.96)	.259	1.45 (1.13–1.87)	.040	58.50%	0.047
Subgroup4: NOS score =6	2	19,23	0.76 (0.42–1.39)	.38	0.76 (0.42–1.39)	.380	0.00%	0.961
NOS score =7	2	20,22	1.79 (1.28–2.50)	.001	1.79 (1.28–2.50)	.001	0.00%	0.639
NOS score =8	3	10,12,25	1.15 (0.61–2.16)	.665	1.29 (0.87–1.93)	.210	53.10%	0.119

N = number of studies, HR = hazard ratio, 95% CI = 95% confidence interval, Ph = p values of Q test for heterogeneity test, OS = Overall survival, DFS = Disease-free survival, NOS score = The Newcastle-Ottawa Scale (NOS) score; NA = not applicable.

### 3.6 Publication bias

Publication bias was not detected implied by Begg plots in this meta-analysis for OS (*P* = .640), also the same result for DFS (*P* = .072) (Fig. 4 ). Egger test also was used, and the results were shown in Supplemental Digital Content, Fig. S4, in which the evidence of publication bias was indicated for OS (*P* = .001) and DFS (*P* = .014) intriguingly.

**Figure 4:**
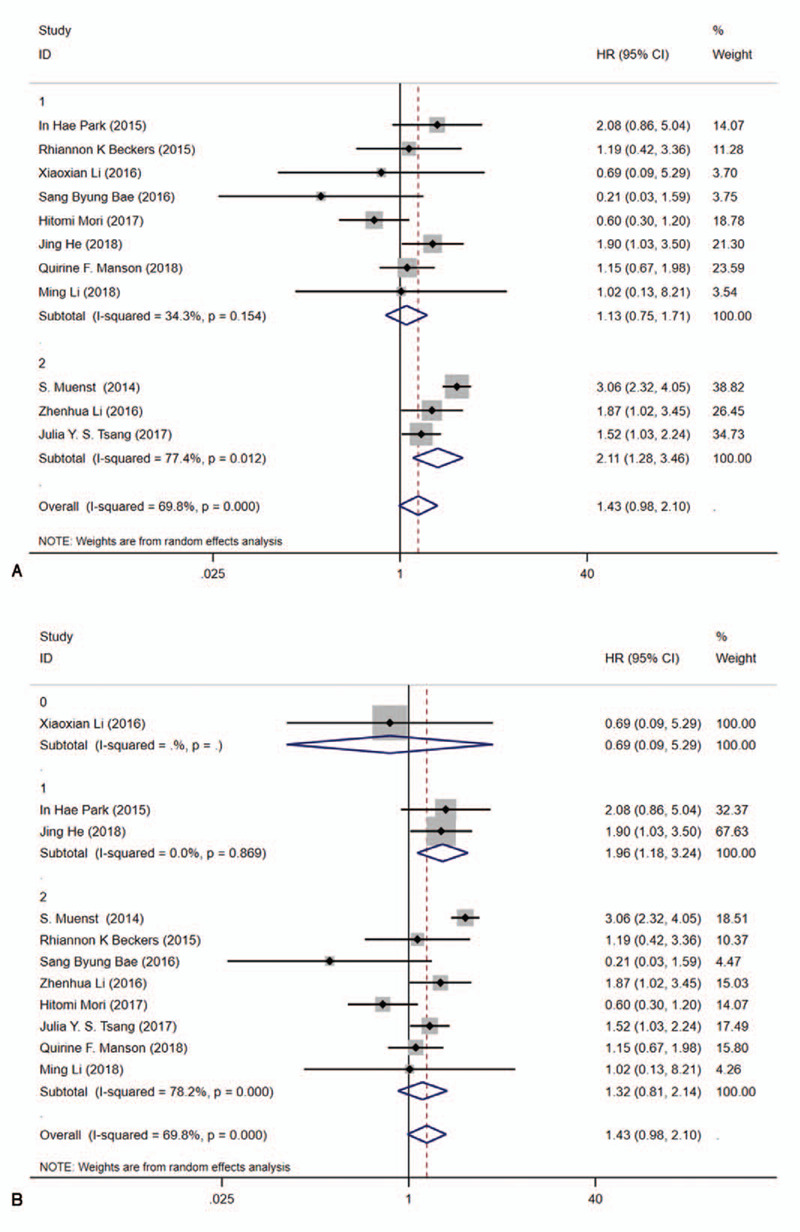
Forest plots for the association between PD-L1 expression and literature heterogeneity factors of OS with a random model. A, sample size (≤500 and >500). B age (≤50 and >50). C, Univariate analysis and multivariate analysis(U/M). D, NOS score.^[6–8]^

**Figure 4 (Continued):**
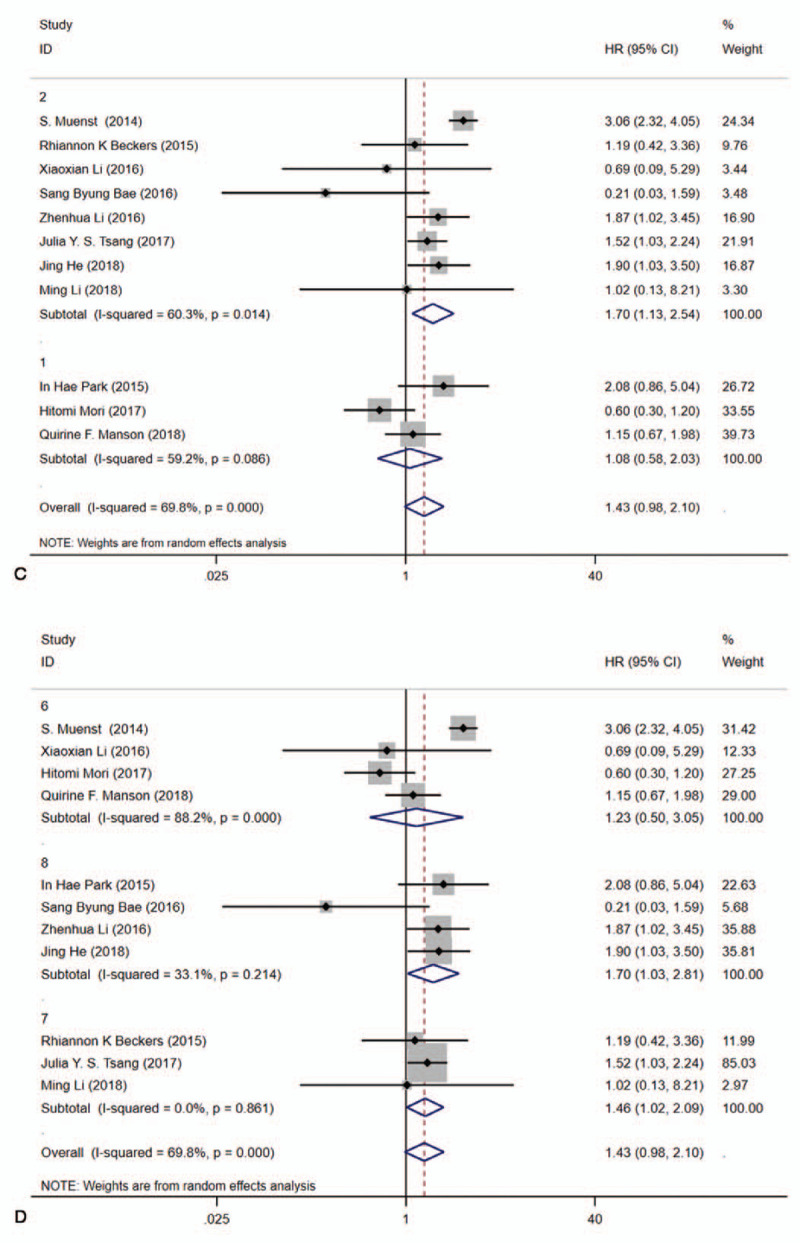
Forest plots for the association between PD-L1 expression and literature heterogeneity factors of OS with a random model. A, sample size (≤500 and >500). B age (≤50 and >50). C, Univariate analysis and multivariate analysis(U/M). D, NOS score.^[6–8]^

## 4 Discussion

Although some clinical studies have reported the effect of PD-L1 expression on BC patients, its role is still uncertain.

With the advancement of technologies, tumor identifying and treatment have been improved and benefit has been achieved for patients with BC, meanwhile, the immunotherapy also helps the therapy progression and drug regulation in terms of patient survival.^[28]^ Recently, research indeed discovered quite a few molecules as prognosis biomarkers for BC. Within the great performance of biomarkers inhibitors, properties such as tumor invasion and aggressiveness can be eased to some extent, which may also associate with survival features or events that are exactly analyzed in this study.^[29]^ That PD-L1 expression related to poor prognosis was confirmed by some previous researches.^[30]^ PD-1 and PD-L1 pathway not only contributes a lot to immune microenvironment, also meaningfully effects on various tumors like lung cancer and colon cancer,^[31–35]^ malignant melanoma,^[36]^ and tumors of gynecology,^[37]^ urinary malevolent tumors.^[38]^

PD-L1 expression also has been analyzed by several previous cohorts in patients with BC, which found that the correlation is still controversial. Meanwhile, clinical trials demonstrated that the PD-L1 inhibitors are able to improve the prognostic events for patients.^[33,39]^ Our data and results showed that it indeed meets the founding we expect. The mechanism about the binding of PD-L1 in tumor cells and PD-1 on T cells was confirmed to facilitate tumor immune escape, resulting in cancer immune tolerance.^[40]^

High tumor stage, lymph node metastasis, ER negativity, HER2 positivity, luminal B and TNBC molecular subtype, and Ki-67 high expression were found to catch eyes considering prognostic factor as for survival with 12 studies including 4336 patients. Meanwhile, these results were in agreement with several lines of evidence that support the immunogenicity of TNBC: than other subtypes, expression of PD-L1 in level of mRNA in TNBC is evidently higher.^[41]^ The correlation between PD-L1 and these clinical prognostic molecules had also been mentioned in other studies,^[42–44]^ although the results are not exactly the same, suggesting the close relationship between PD-L1 and BC clinical patients. However, our study strictly formulated inclusion and exclusion criteria, screened out all BC patients treated with radiotherapy and chemotherapy or other drugs before and after surgical treatment in which including different and novel studies, and more accurately described the prognostic role of PD-L1 in BC patients.

In this meta-analysis, compared to patients with PD-L1 negative expression, PD-L1 positivity associated with worse OS (HR:1.43; 95% CI:0.98–2.10; *P* < .001)and might have no obvious tight connection with DFS (HR:1.40; 95% CI:1.11–1.78; *P* = .101) and RFS (HR:2.36; 95% CI:1.04–5.34; *P* = .145). Subgroup analyses revealed that sample size of individual study may explain the heterogeneity of the shorter DFS by PD-L1 expression, and factors such as age, Univariate/multivariate, and literature quality might be not responsible for heterogeneous root of prognosis. Besides, the lack of standardization for detection could also be responsible for the discrepant turnout due to the multiple using of TMAs within IHC for PD-L1 in nearly half of studies.^[9,10,18–21,24–26]^ To overcome the above shortcomings, whole-tissue sections would be a good option.

Resulting from PD-L1 positive linked with shorter survival events considering various epithelial-originated cancers, various studies tended to treat it as a novel prognostic marker.^[45]^ However, there also appeared conflicting result unsurprisingly.^[46,47]^ The exact mechanism between immune microenvironment and tumor was undefined as we know. Thus, the correlation between immune microenvironment and tumor development has been increasingly urgent to explore and summary, especially about survival events. Meta-analysis is common tool to collect huge data and summary controversial events whereas there were still several limitations. Due to retrospective studies, some bias would be considered. Besides, this analysis was constrained to pure English studies, causing some specific ethics data group were excluded subconsciously. the origin of heterogeneity cannot be fully traced still even the sensitivity and subgroup analyses were conducted. Meanwhile, even though Begg plots showed that publication bias did not exist, however, classic funnel plot showed obvious asymmetry (Supplemental Digital Content Fig. S5), which was further confirmed with the Egger test (*P* < .05). After using the trim-and-fill method, for OS random effects pooled HRs was adjusted to 1.430 (95%CI:0.976–2.096) and for DFS pooled HRs was adjusted to 1.402 (95%CI 1.107–1.776). Although this study owns strict inclusion and exclusion criteria, it also loses a lot of valuable literature, resulting in relatively small eligible studies in the establishment of prognostic values, which should be poured attention into.

## 5 Conclusions

Higher tumor stage, lymph node metastasis, ER negativity, HER2 positivity, luminal B and TNBC molecular subtype, and Ki-67 high expression were found to be related to poorer OS in BC. Positive PD-L1 expression may be meaningful to some degree for predicting prognosis events in BC, which needs to be explored and verified by other large-scale researches.

## Acknowledgments

The authors would like to acknowledge the efforts of each individual that enrolled in the same team of professor Liu of Department of Endocrine Breast Surgery, The First Affiliated Hospital of Chongqing Medical University.

## Author contributions

**Conceptualization:** Yingzi Zhang.

**Data curation:** Yingzi Zhang, Jiao Tian, Chi Qu.

**Methodology and Formal analysis:** Zhenrong Tang, Yu Wang, Kang Li.

**Supervision:** Yuan Yang.

**Writing – original draft:** Yingzi Zhang, Yuan Yang.

**Writing – review & editing:** Yingzi Zhang, Jiao Tian, Chi Qu.

## Supplementary Material

Supplemental Digital Content

## Supplementary Material

Supplemental Digital Content

## Supplementary Material

Supplemental Digital Content

## Supplementary Material

Supplemental Digital Content

## Supplementary Material

Supplemental Digital Content
