# Supplementary material for: Prognostic value of programmed cell death ligand-1 expression in breast cancer: A meta-analysis
Source: Medicine (Baltimore). 2020 Dec 4;99(49):e23359. doi: 10.1097/MD.0000000000023359 (PMC7717727; doi:10.1097/MD.0000000000023359)

**Prognostic value of programmed cell death ligand-1 expression in breast cancer: A systematic review and meta-analysis**

Yingzi Zhang^1^, MM, JiaoTian^1^, MM, Chi Qu^1^, MM, Zhenrong Tang^1^, MM, YuWang^1^, MM, Kang Li^1^, MM, YuanYang^2^, MD, PhD, Shengchun Liu^1^*, MD, PhD.

# ^1^Department of Endocrine Breast Surgery, The First Afﬁliated Hospital of Chongqing Medical University, Chongqing, China

# ^2^ Department of Cardiovascular Medicine, the First Affiliated Hospital of Chongqing Medical University, Chongqing, China

# * Corresponding author: Shengchun Liu

# Email: [liushengchun1968@163.com](mailto:liushengchun1968@163.com)

**Supplemental Digital Content (Figure S1) Sensitivity analysis.** No individual study significantly influenced the overall HRs in which one study was removed at a time. **A** OS. **B** DFS.

(A)
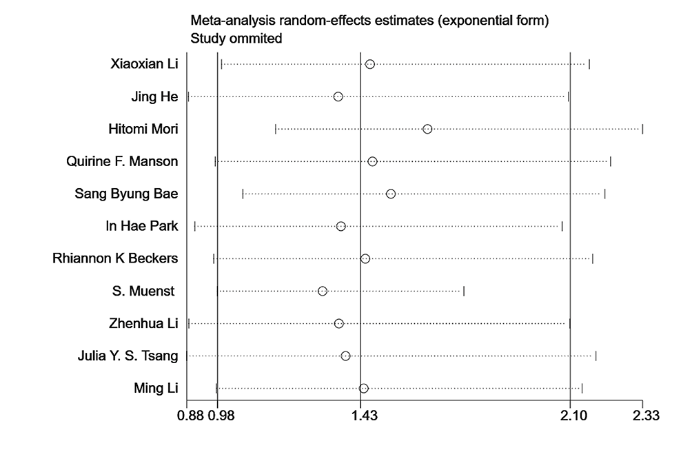


(B)
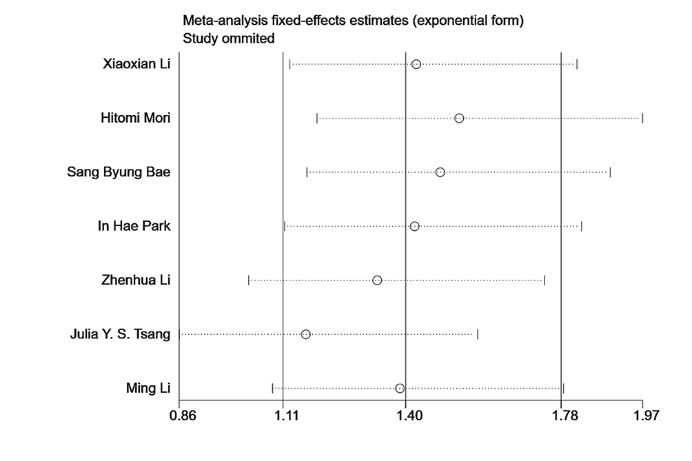

Supplement: Supplemental Digital Content [file medi-99-e23359-s001.docx]
